# Supplementary material for: Construction of an anthropometric discriminant model for identification of elite swimmers: an adaptive lasso approach
Source: PeerJ. 2023 Jan 9;11:e14635. doi: 10.7717/peerj.14635 (PMC9835708; doi:10.7717/peerj.14635)

1 Stature

[Definition] The vertical distance from the top point of the head to the ground when the human body is standing up.
[Equipment] Height / sitting height meter.
[Preparation] Subjects should be crown-free, barefoot and untie the hair bun when measuring.
[Method] The subject should stand on the base plate in an upright posture (upper limbs naturally drooping, heels together, and toes 60 degrees apart). The heel, sacrum and shoulder blades are close to the column, the trunk is naturally straight, the head is upright, the eyes are looking straight ahead, and the upper edge of the ear-screen is level with the lower edge of the eye-socket. The tester stands on the right side of the subject and gently slides the horizontal pressure plate down, pressing it loosely and appropriately on the top of the subject's head, with both eyes parallel to the horizontal pressure plate for reading. When recording, the unit is centimeter (cm), retain one decimal, and allow an error of 0.5 cm.
[Notice]
①The height / sitting height meter should be placed in a flat place against the wall, with the scale of the column facing the light source.
②When measuring, pay attention to the heel, sacrum and between both scapulae, these three points are close to the column; under the premise of ensuring that the upper edge of the ear-screen is level with the lower edge of the eye-socket, the back side of the head is as close to the column as possible.
③When the horizontal pressure plate is in contact with the top of the head, the degree of elasticity should be moderate, and women wearing hairpins that affect the measurement should be removed.
④When reading, the tester's eyes are at the same level as the bottom of the slide test plate, and read the value indicated by the corresponding column on the bottom of the slide test board.

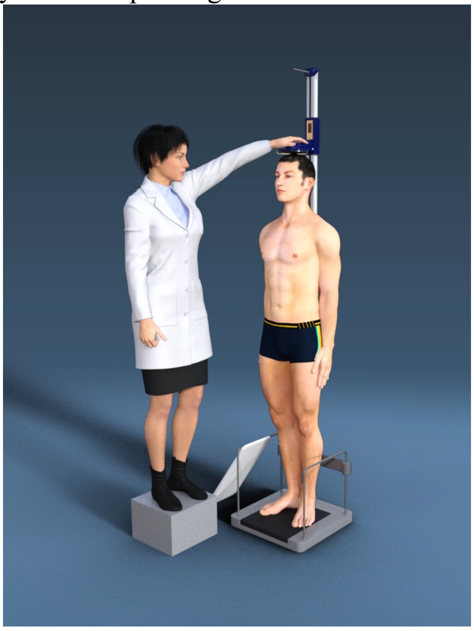

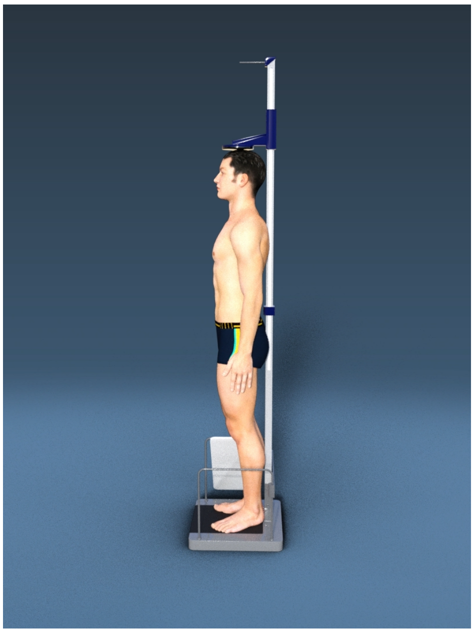

2 Sitting height

[Definition] The vertical distance from the highest point on the median line of the head to the seat plane when the human body is sitting.
[Equipment] Height / sitting height meter.
[Preparation] Subjects should be crown-free and untie the hair bun when measuring.
[Method] The subject should bend down and sit on the seat plate after bringing the sacrum close to the column, so that the sacrum and the two scapulae are close to the column. The torso and head are upright, the eyes are looking straight ahead, and the upper edge of the ear-screen is level with the lower edge of the eye-socket. The legs are together, with the thighs parallel to the ground and at right angles to the calves. The upper limbs hang naturally, the palms of the hands do not support the seat plate, and the feet are flat on the ground or on the mat. The tester stands on the right side of the person being tested, slides the horizontal pressure plate gently and presses it loosely at the top of the person's head with both eyes parallel to the

horizontal pressure plate for reading. When recording, the unit is centimeter (cm), retain one decimal, and allow an error of 0.5 cm.

[Notice]

①The height / sitting height meter should be placed on a flat surface, and the column should be against the wall.

②The subject's sacrum and between both scapulae should be close to the column, and women wearing hairpins that affect the measurement should be removed.

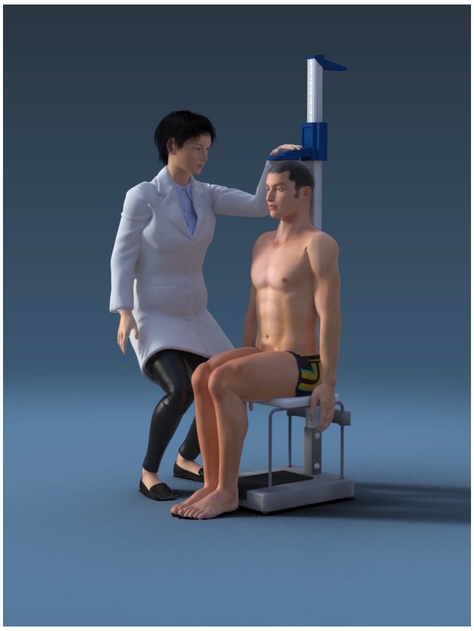
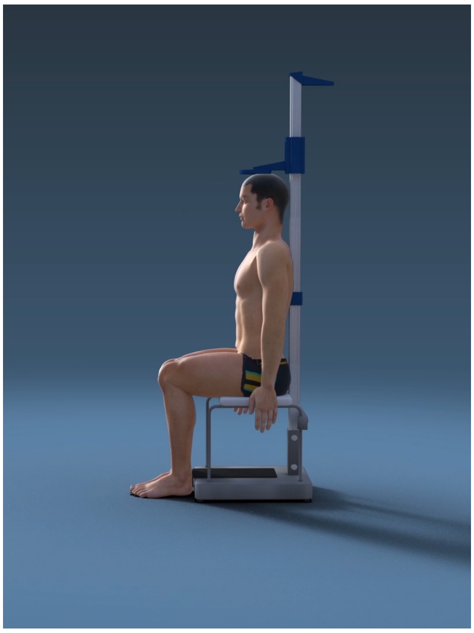

3 Span

[Definition] The maximum straight-line distance between the ends of two middle fingers (excluding the nail) when in the horizontal position.

[Equipment] Digital display Martin ruler (Martin-CHN).

[Preparation] Subjects should wear as little clothing or underwear as possible on the upper body when measuring.

[Method] The ruler is fixed on the platform, the subject's feet are separated, the two arms are raised laterally, the upper body is lying on the ruler, the end of the middle finger of one upper limb is fixed in the zero position of the ruler, the other upper limb is stretched to the side as far as possible, and the two arms become a straight line. The tester observes the distance between the tips of the subject's two middle fingers (excluding the nails) and then reads and records the value. When recording, the unit is centimeter (cm), retain one decimal, and allow an error of 0.5 cm.

[Notice]

①The subject's arms and sternum are pressed against the table and the ruler, and the upper limbs are fully extended to the other side as far as possible, with both arms in a straight line.

②The end of the middle finger of one hand of the subject is fixed on the zero position of the ruler must not move.

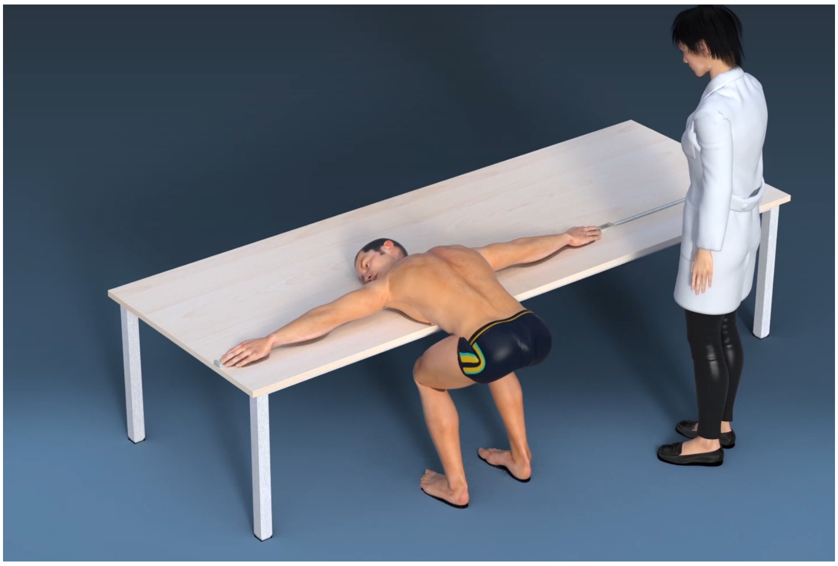

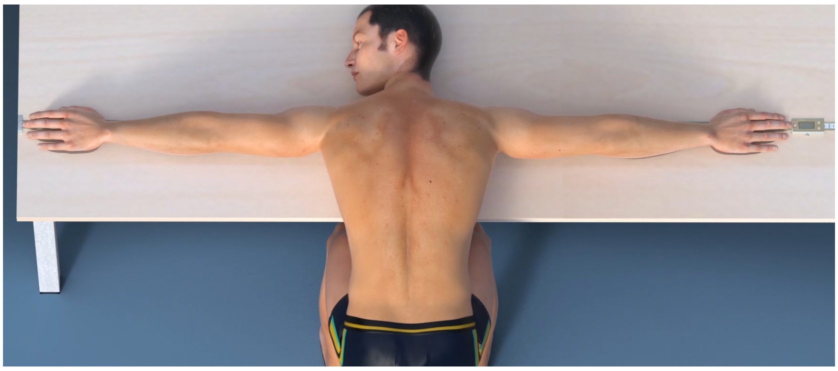

4 Upper limb length

[Definition] The vertical distance from the point of the shoulder peak to the end of the middle finger (excluding the nail).

[Equipment] Digital display Martin ruler (Martin-CHN).

[Preparation] The subject's upper body is less clothed during the measurement.

[Method] The subject stands naturally with both feet apart, shoulder-width apart, both arms naturally hanging straight down, and both eyes looking straight ahead. The tester stands on the subject's right side and measures the vertical distance from the peak of the shoulder to the end point of the middle finger (excluding the nail), read and record the value. When recording, the unit is centimeter (cm), retain one decimal, and allow an error of 0.5 cm.

[Notice]

①The subject's shoulders are flush, not one high and one low, and the upper limbs are down as straight as possible.

②The subject's palms, fingers and forearms must be kept straight, fingers cannot be cocked.

③The ruler should be vertical to the ground.

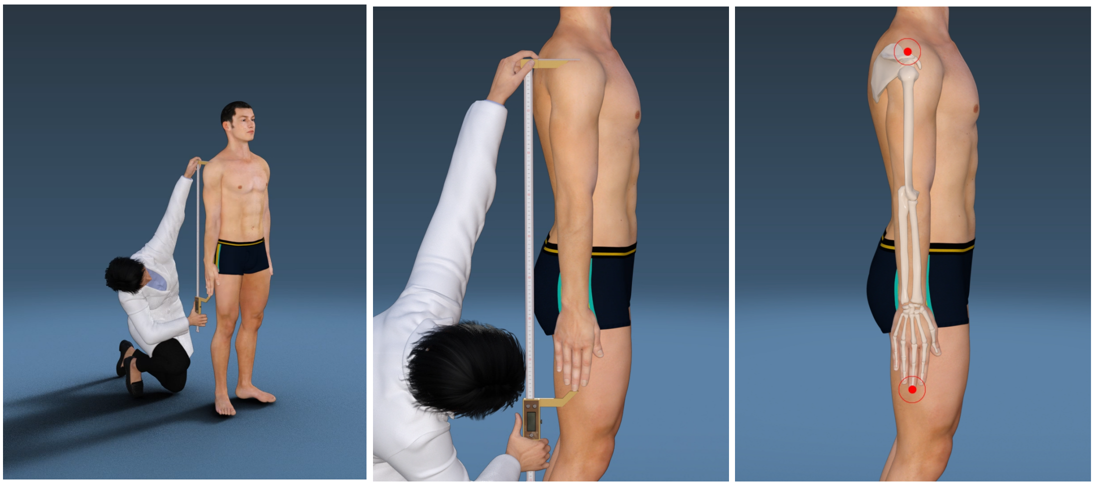

5 Upper arm length

[Definition] The vertical distance from the point of the shoulder peak to the point of the lateral epicondyle of the humerus.
[Equipment] Digital display Martin ruler (Martin-CHN).
[Preparation] The subject's upper body is less clothed, and the upper arm is bare during the measurement.
[Method] The subject stands naturally with both feet shoulder-width apart and the arm straight, the tester stands on the subject's right side and measures the distance between the shoulder peak point and the point of the lateral epicondyle of the humerus. When recording, the unit is centimeter (cm), retain one decimal, and allow an error of 0.2 cm.
[Notice]
①Do not shrug your shoulders and neck when measuring, keep both shoulders at the same level.
②The subject's palm, fingers and forearm must be kept in a straight line.

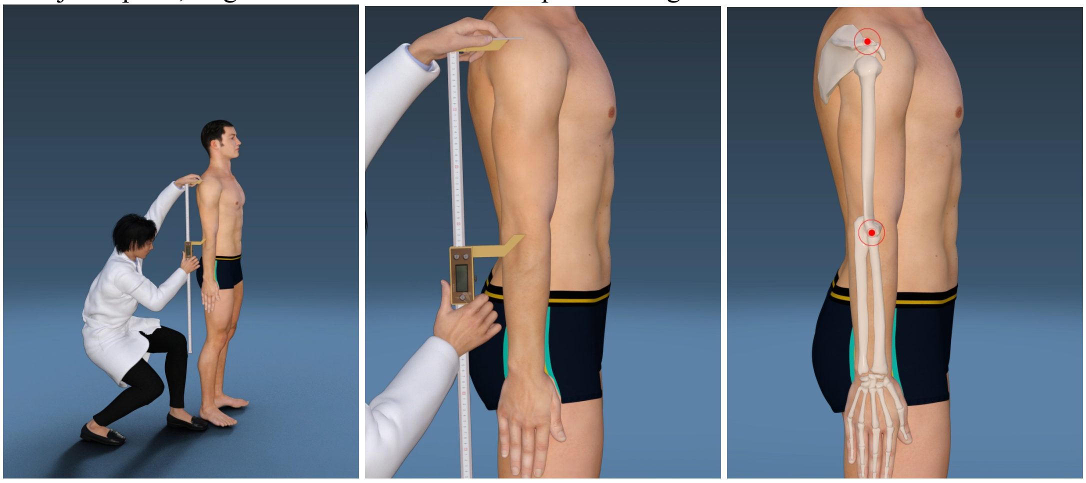

6 Forearm length

[Definition] The straight-line distance from the radial point to the radial stem point.
[Equipment] Digital display Martin ruler (Martin-CHN).
[Preparation] The subject's upper body is less clothed, and the forearm is bare during the measurement.
[Method] The subject stands with both feet shoulder-width apart, both arms straight down, and the right hand with five fingers together. The tester stands on the subject's right side, the fixed end of the ruler (zero position) is aligned with the radial point, and the vernier is moved to the radial stem point for reading. When recording, the unit is centimeter (cm), retain one decimal, and allow an error of 0.2 cm.
[Notice]
①No shrugging of the shoulders and neck when measuring, so that the two shoulders remain at the same

level.
②The subject's upper limbs must be in a straight line, and the position of the two points should be found correctly.

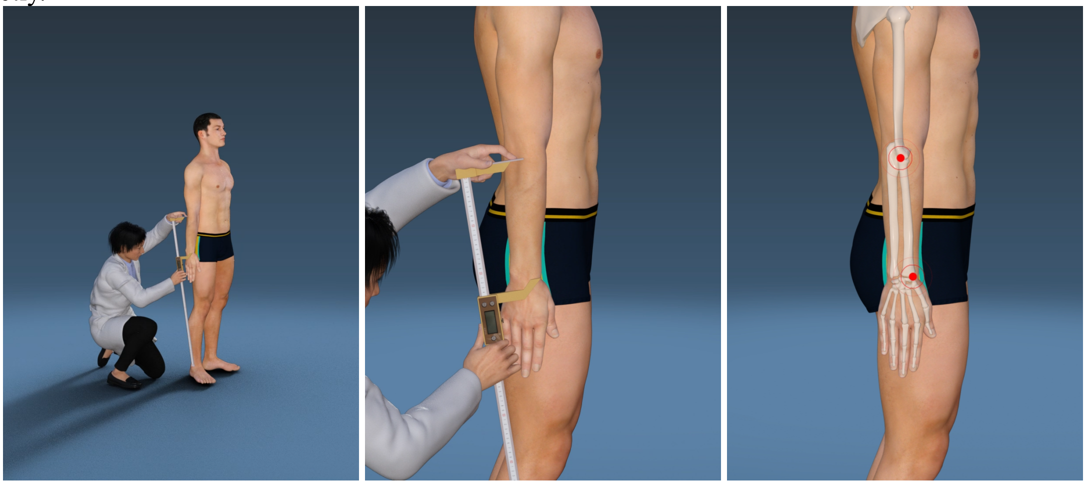

7 Acromion height

[Definition] The vertical distance from the shoulder peak point to the ground.
[Equipment] Infrared digital measuring instrument.
[Preparation] The subject is barefoot during the measurement, with as little clothing as possible on the upper body.
[Method] The subject stands with both feet shoulder-width apart, and the right arm is naturally straight down with the five fingers together. The tester stands behind the subject's right side, fixes one end of the measuring instrument at the shoulder peak point so that the infrared irradiation point falls vertically on the ground, and then presses the infrared switch to measure the vertical distance from the shoulder peak point to the ground. When recording, the unit is centimeter (cm), retain one decimal, and allow an error of 0.5 cm.
[Notice]
①The ground should be flat, the muscles of the lower limbs should be relaxed when standing, both shoulders should be kept horizontal, and the spine should not be bent. The body weight should fall equally on both feet.
②The distance between the laser point and the outside of the foot is about 6 cm, at which point it is stated that the laser beam is basically perpendicular to the ground.

8 Hand length

[Definition] The straight-line distance from the midpoint of the transverse wrist line to the tip point of the middle finger (excluding the nail).
[Equipment] Digital Martin ruler (Martin-CHN).
[Preparation] The subject takes off the bracelet or watch.
[Method] The subject's right hand is extended forward with the palm up, the five fingers together and straight, and the palm of the hand is in a straight line with the forearm. Measure the straight-line distance from the midpoint of the transverse wrist line of the distal radius and ulna to the tip of the middle finger (excluding the nail). When recording, the unit is centimeter (cm), retain one decimal, and allow an error of 0.2 cm.
[Notice]
①The subject's palm and forearm should be flat on the table, the hand should be fully extended, the fingers should not be cocked, and the tip of the middle finger without the nail should be measured.
②The determination of the distal carpal transverse line of the radius and ulna can be measured by using the thumb and index finger of one hand to hold the distal carpal joint suture of the radius and ulna stem point, and taking the midpoint of the skin wrinkle that coincides with it, basically taking the first transverse line of the nearest end of the carpal palm as the benchmark.

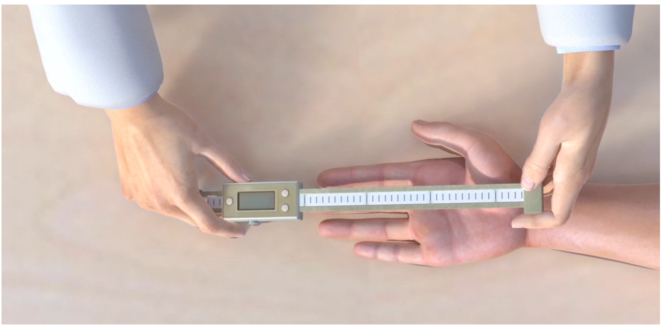
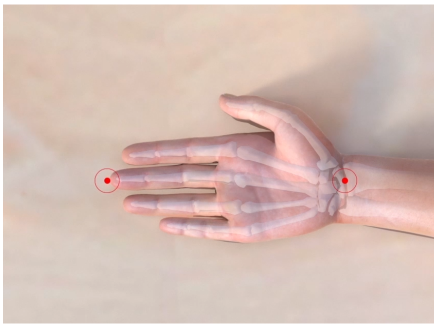

Supplement: Supplemental Information 2 — Includes stature, sitting height, span, upper limb length, upper arm length, forearm length, acromion height, and hand length. [file peerj-11-14635-s002.pdf]
