# Supplementary material for: Construction of an anthropometric discriminant model for identification of elite swimmers: an adaptive lasso approach
Source: PeerJ. 2023 Jan 9;11:e14635. doi: 10.7717/peerj.14635 (PMC9835708; doi:10.7717/peerj.14635)
Supplement: Supplemental Information 3 — Includes iliospinale posterior height, trochanterion height, gluteal height, eristailiaca height, thigh length, lower leg length, achilles tendon length, foot length, and back of foot height. [file peerj-11-14635-s003.pdf]

9 Iliospinale posterior height

[Definition] The vertical distance from the point of anterior superior iliac spine to the ground.
[Equipment] Digital display Martin ruler (Martin-CHN).
[Preparation] The subject should be barefoot and wear sports shorts during the measurement.
[Method] The subject stands with both feet shoulder-width apart, the knee is straight, natural standing, the tester stands on the subject's left side, and leg length A measures the vertical distance between the left anterior superior iliac spine point and the standing surface. When recording, the unit is centimeter (cm), retain one decimal, and allow an error of 0.5 cm.
[Notice]
①The ground should be flat, the muscles of the lower limbs should be relaxed when standing, and the weight should fall evenly on both feet.
②Ruler shall be perpendicular to the support surface.

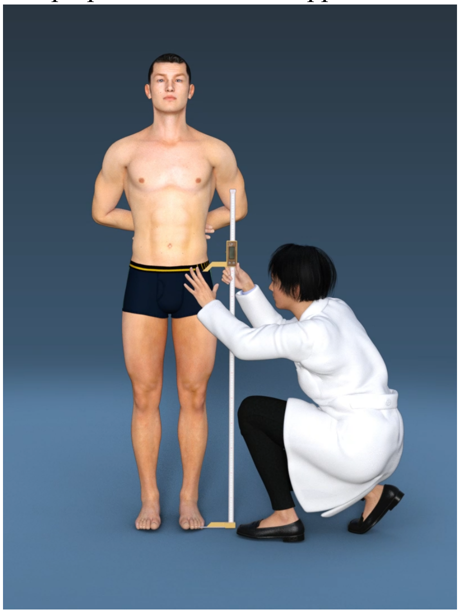

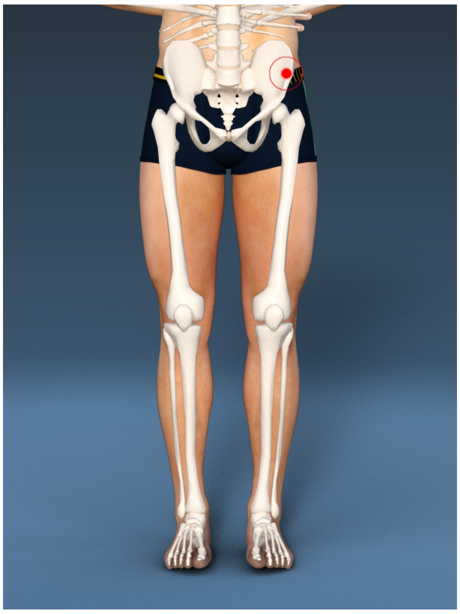

10 Trochanterion height

[Definition] The vertical distance from the highest point of the trochanter of the femur to the ground.
[Equipment] Digital Martin ruler (Martin-CHN).
[Preparation] The subject should be barefoot and wear sports shorts during the measurement.
[Method] The subject stands with both feet shoulder-width apart, the knee is straight, natural standing, the tester stands on the subject's left side, and the leg length B measures the vertical distance from the trochanter of the left femur to the standing surface. When recording, the unit is centimeter (cm), retain one decimal, and allow an error of 0.5 cm.
[Notice]
①The ground should be flat, the muscles of the lower limbs should be relaxed when standing, and the weight should fall evenly on both feet.
②Ruler shall be perpendicular to the support surface.

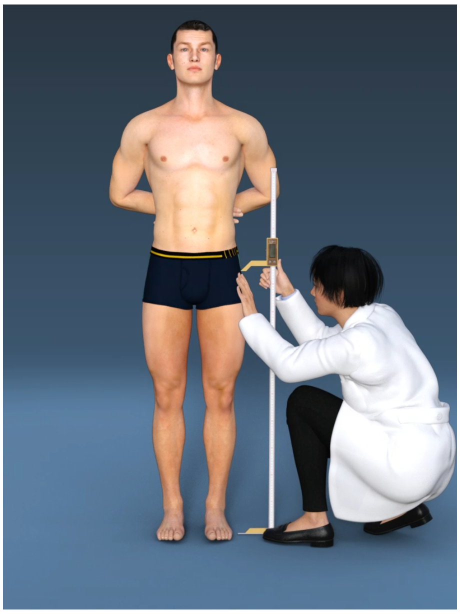

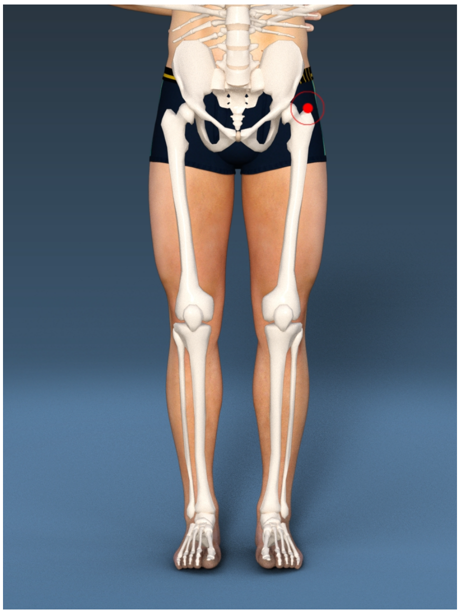

11 Gluteal height

[Definition] The vertical distance from the lowest point of the hip line to the ground.
[Equipment] Digital display Martin ruler (Martin-CHN).
[Preparation] The subject should be barefoot and wear sports shorts during the measurement.
[Method] The subject stands with both feet shoulder-width apart, the knee is straight, natural standing, and the leg length C is measured with the tester stand behind the subject so that the hip line between the hip and thigh is exposed, align the cursor horizontally with the lowest point of the hip line and read the vertical distance from the hip line to the ground. When recording, the unit is centimeter (cm), retain one decimal, and allow an error of 0.5 cm.
[Notice]
①The ground should be flat, the muscles of the lower limbs should be relaxed when standing, and the weight should fall evenly on both feet.
②Ruler should be perpendicular to the support surface.
③When measuring the leg length C, it is recommended that the test be performed in a private room, with the subject wearing shorts and lifting the lower edge of the shorts appropriately so that the hip line can be clearly observed.

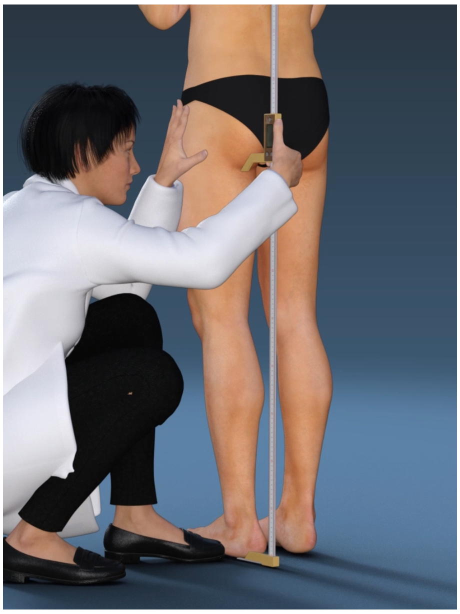

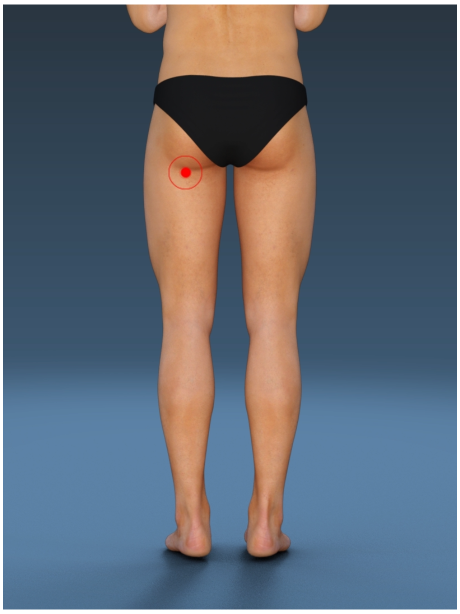

12 Eristailiaca height

[Definition] The vertical distance from the point of iliac crest to the ground.
[Equipment] Digital display Martin ruler (Martin-CHN).
[Preparation] The subject should be barefoot and wear sports shorts during the measurement.
[Method] The subject stands with both feet shoulder-width apart, the knee is straight, natural standing, the tester stands on the subject's left side, the leg length H measure the vertical distance from the intersection of the left iliac crest and axillary midline to the standing surface. When recording, the unit is centimeter (cm), retain one decimal, and allow an error of 0.5 cm.
[Notice]
①The ground should be flat, the muscles of the lower limbs should be relaxed when standing, and the weight should fall evenly on both feet.
②Ruler should be perpendicular to the support surface.
③When measuring the leg length H, the subject's pelvis should be kept horizontal and not tilted.

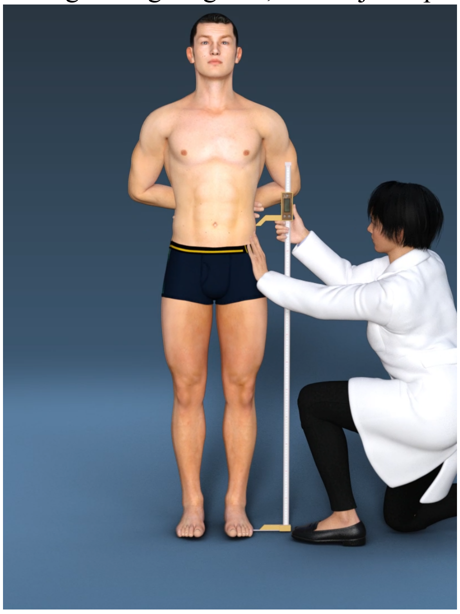

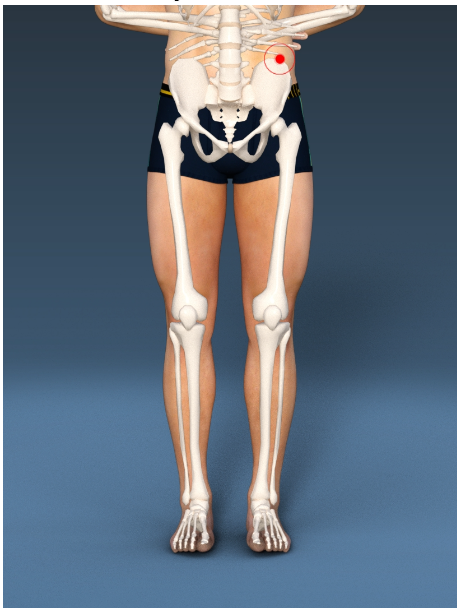

13 Thigh length

[Definition] The distance between the point of the anterior superior iliac spine and the point of the tibia.
[Equipment] Digital display Martin ruler (Martin-CHN).
[Preparation] The subject should be barefoot and wear sports shorts during the measurement.
[Method] The subject's feet were separated, knees were straightened, and the subject was standing naturally, the tester stands on the subject's left side, align the fixed end of the ruler with the anterior superior iliac spine point, then move the ruler's vernier to the tibial point and measure the distance between the anterior superior iliac spine point and the tibial point. When recording, the unit is centimeter (cm), retain one decimal, and allow an error of 0.5 cm.
[Notice]
①The ground should be flat, the muscles of the lower limbs should be relaxed when standing, and the weight should fall evenly on both feet.
②The two bony marks of the anterior superior iliac spine point and the tibial point should be found correctly.

14 Lower leg length

[Definition] The vertical distance from the upper edge of the medial tibial condyle to the support surface.
[Equipment] Digital display Martin ruler (Martin-CHN).
[Preparation] The subject should be barefoot and wear sports shorts during the measurement.
[Method] The subject should stand, lift the left leg, bend the knee and step on the stool surface with the full foot steadily, with the lower leg perpendicular to the stool surface. Ruler is placed vertically on the inner

side of the measuring leg. The tester is positioned on the right side of the measuring leg and touches and measures the vertical distance from the upper edge of the medial condyle of the subject's left tibia (the point of the tibia at the knee gap) to the bench surface. When recording, the unit is centimeter (cm), retain one decimal, and allow an error of 0.5 cm.

[Notice]

①The medial tibial epicondyle point should be found correctly, the tester can open the right hand to hold the knee joint at the same time, the inside of the thumb along the knee joint seam to touch the uppermost part of the inner calf, then the point can be touched.

②The calf should be perpendicular to the ground and the ruler should be parallel to the tibia.

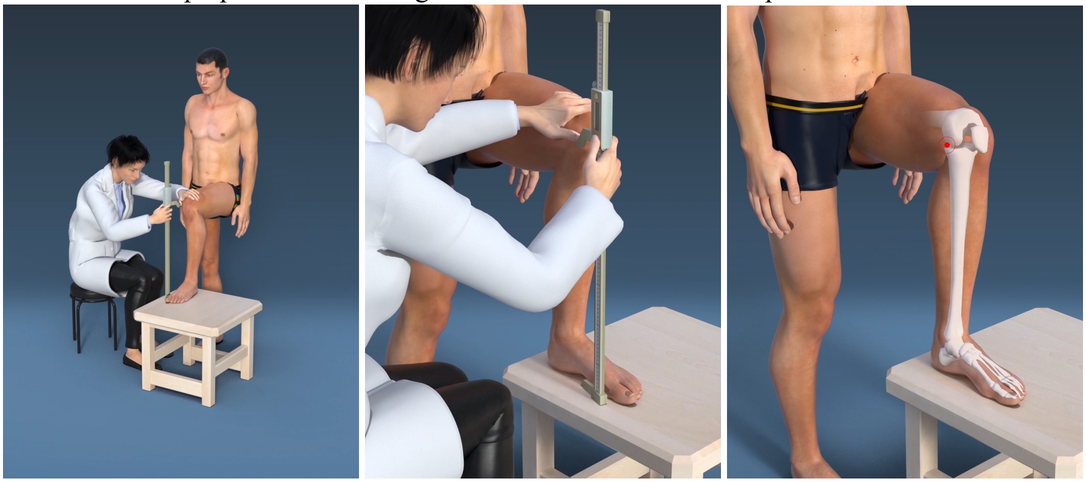

15 Achilles tendon length

[Definition] The straight-line distance between the lower edge of the medial gastrocnemius muscle belly and the heel node.

[Equipment] Digital display Martin ruler (Martin-CHN).

[Preparation] The subject should be barefoot and wear sports shorts during the measurement.

[Method] The subject stands facing the wall, with the legs naturally separated and both hands holding the wall and lifting the heel, so that the calf triceps muscle is fully contracted. Tester draw a short mark line in the subject's lower edge of the left leg gastrocnemius muscle medial muscle belly, and then let the subject return to a natural standing posture, with the fixed end of the ruler (zero scale point) aligned with the heel point, move the vernier to the lower edge of the muscle belly mark line, read and record. When recording, the unit is centimeter (cm), retain one decimal, and allow an error of 0.2 cm.

[Notice]

①Calf triceps muscle must be fully contracted, so that the gastrocnemius shape fully revealed.

②Do not move the skin when drawing the mark.

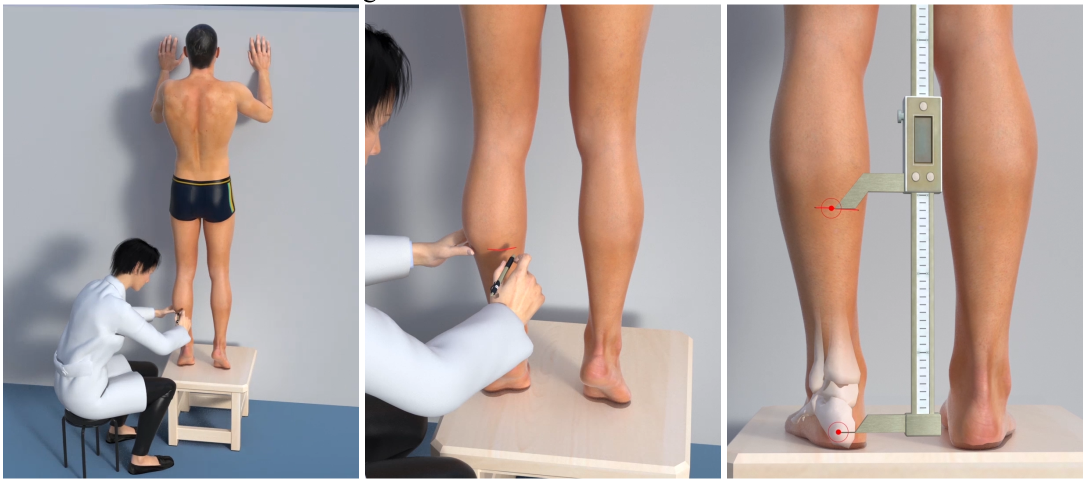

16 Foot length

[Definition] The maximum linear distance from the point of the heel to the point of the toe (excluding the nail).
[Equipment] Foot length / breadth measuring tape.
[Preparation] The subject should be barefoot.
[Method] The subject places the left foot on the measuring tape, the heel against the rear stopper, the sole of the foot is stretched and flat, the foot parallel to the horizontal scale, push the front of the slide, measure the distance between the point of the heel to the point of the longest toe. When recording, the unit is centimeter (cm), retain one decimal, and allow an error of 0.2 cm.
[Notice]
①The foot must be fully stretched and flat when measuring.

17 Back of foot height

[Definition] The vertical distance from the anterior inferior point of the tibia to the support surface.
[Equipment] Digital display Martin ruler (Martin-CHN).
[Preparation] The subject should remove shoes and socks and be barefoot during the test.
[Method] The subject stands with the left leg raised, bends the knee and places the foot on the stool surface, with the full foot on the stool surface and the lower leg perpendicular to the stool surface, and measures the vertical distance from the lower point of the tibia to the stool surface. When recording, the unit is centimeter (cm), retain one decimal, and allow an error of 0.2 cm.
[Notice]
①The lower leg must be placed perpendicular to the horizontal plane.
②The foot must be completely stretched and flat when measuring.
