# Supplementary material for: Construction of an anthropometric discriminant model for identification of elite swimmers: an adaptive lasso approach
Source: PeerJ. 2023 Jan 9;11:e14635. doi: 10.7717/peerj.14635 (PMC9835708; doi:10.7717/peerj.14635)
Supplement: Supplemental Information 4 — Includes biacromial breadth, biiliocristal breadth, bitrochanteric breadth, hand breadth, foot breadth, biepicondylar humerus breadth, and biepicondylar femur breadth. [file peerj-11-14635-s004.pdf]

18 Biacromial breadth

[Definition] The distance between the outer edge of the two shoulder peak points.
[Equipment] Digital display Martin ruler (Martin-CHN).
[Preparation] The upper body is less clothed or wears only close-fitting underwear during measurement.
[Method] The subject stands with both feet shoulder-width apart, and both shoulders relaxed. The tester stands behind him/her and first uses the index finger of both hands to feel outward along the scapular post to the shoulder peak point, then uses ruler to measure the distance between the outer edges of the two shoulder peak points. When recording, the unit is centimeter (cm), retain one decimal, and allow an error of 0.2 cm.
[Notice]
①The subject should relax both shoulders naturally, both shoulders should be at the same height, not to lower the head, contain the chest, shrug the shoulders, or hold the chest.
②The ruler should be kept under the horizontal position when measuring, and the tightness should be appropriate.

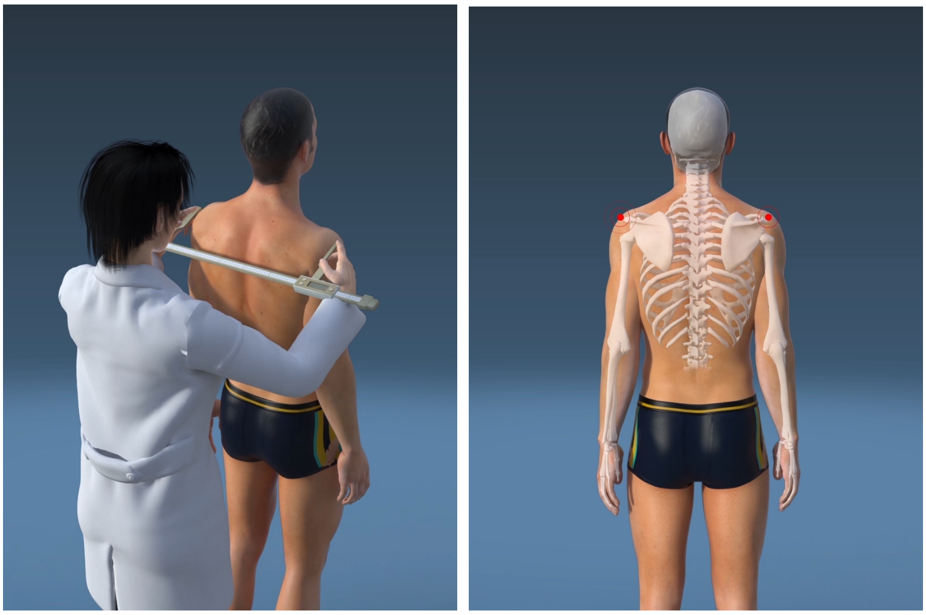

19 Biiliocristal breadth

[Definition] The distance between the outer edge of the two iliac crest points.
[Equipment] Digital display Martin ruler (Martin-CHN).
[Preparation] The upper body is less clothed or wears only close-fitting underwear during the measurement.
[Method] The subject stands naturally with both feet together. The tester faces the subject and touches the iliac crest point (the most outwardly protruding point of the iliac crest) with the index finger of each hand and measures the distance between the outer edges of the two iliac crest points. The distance between the two iliac crest points is measured in centimeters (cm), with one decimal retained, and an error of 0.2 cm is allowed.
[Notice]
①The subject should have an even weight on both legs when measuring the iliac width, and the weight should fall evenly on both feet to avoid pelvic tilt.
②The subject shoulders should be naturally relaxed and at the same height, not to lower the head, contain the chest, shrug the shoulders, or hold the chest.
③The ruler should be kept in a horizontal position during measurement and slid back and forth along the outer edge of the iliac crest to determine the widest point along the upper edge of the pelvis. The loosening and tightening should be appropriate.

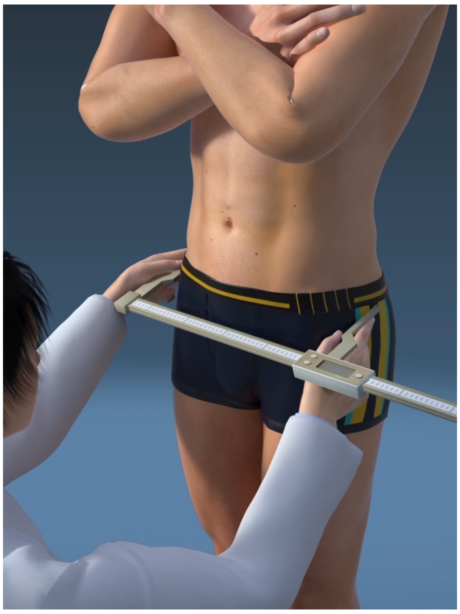 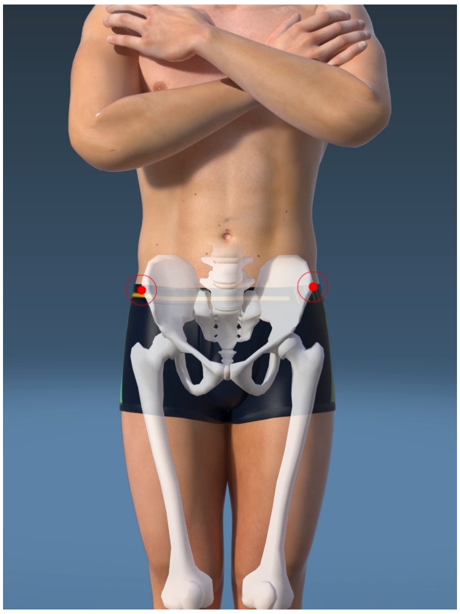

20 Bitrochanteric breadth

[Definition] The straight-line distance between the most outwardly protruding points of the left and right side of the large rotor.
[Equipment] Digital display Martin ruler (Martin-CHN).
[Preparation] The upper body is less clothed or wears only close-fitting underwear when measuring, and the lower body is suitable for wearing sports shorts.
[Method] The subject stands naturally with both feet together, and the tester faces the subject and uses two index fingers to feel the most outwardly protruding point of the right and left femoral trochanter, and measures the distance between the two points. When recording, the unit is centimeter (cm), retain one decimal, and allow an error of 0.2 cm.
[Notice]
①The subject should have an even weight on both legs when measuring the hip width, and the weight should fall evenly on both feet to avoid pelvic tilt.
②The subject shoulders should be naturally relaxed and at the same height, and not to lower the head, contain the chest, shrug the shoulders, or hold the chest.
③The ruler should be kept in a horizontal position when measuring, and the elasticity should be appropriate.

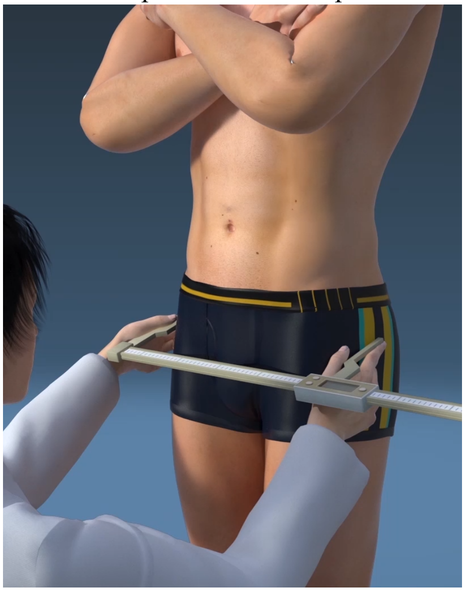 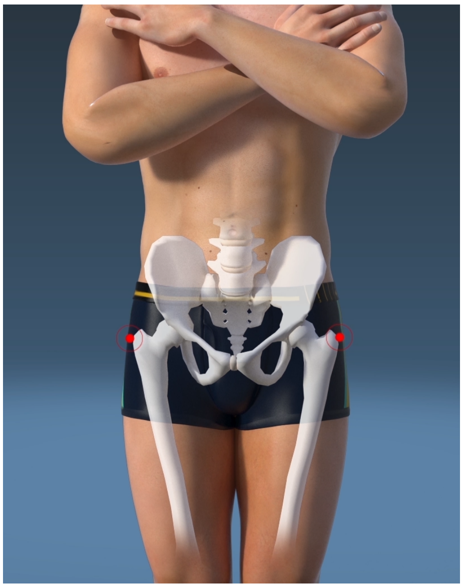

21 Hand breadth

[Definition] The straight-line distance between the radial metacarpal point and the ulnar metacarpal point when the four fingers are straight together.
[Equipment] Digital display Martin ruler (Martin-CHN).
[Preparation] Subjects should cut off the overly long nails.
[Method] The subject extends the right hand forward, palm up, thumbs apart, four fingers together and straight, palm and forearm in a straight line. The tester stands in front of the subject and uses a ruler fixed at the radial metacarpal point and the ulnar metacarpal point to measure the straight line distance between the two points. When recording, the unit is centimeter (cm), retain one decimal, and allow an error of 0.2 cm.
[Notice]
①The hand should be fully straightened when measuring, Ruler should be loose and appropriate, not squeeze the palm, only lightly against the skin, do not have a tight clamp or pressure, the two points position to find the right, pay attention to the difference between the dominant hand.
②Conditions permitting, the subject's palm and forearm should be flat on the table during the test.

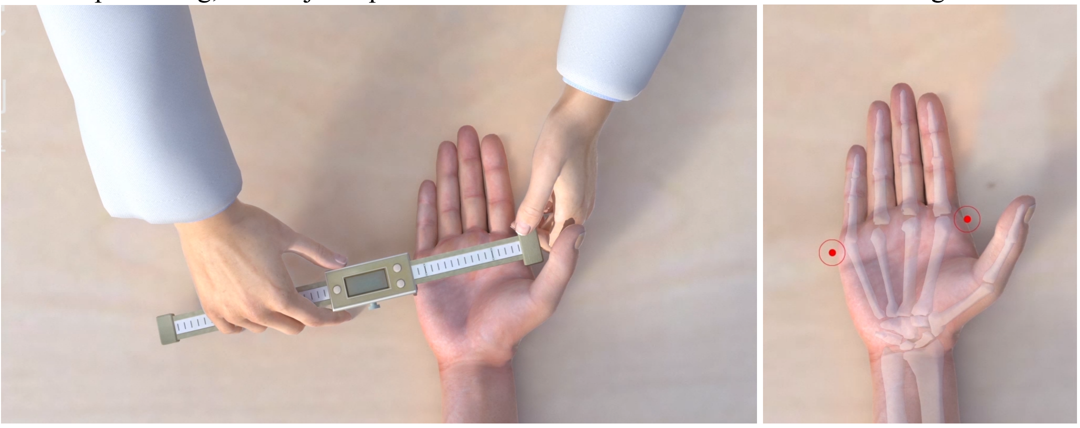

22 Foot breadth

[Definition] The straight-line distance between the tibial metatarsal point and the peroneal metatarsal point.
[Equipment] Digital display Martin ruler (Martin-CHN).
[Preparation] The subject should be barefoot.
[Method] The subject puts the left foot perpendicular to the scale, the widest part of the metacarpal bone is pressed against the base plate of the ruler, the sole of the foot is stretched and flattened, the tester pushes the slide plate and measures the straight line distance of the widest part of the foot. When recording, the unit is centimeter (cm), retain one decimal, and allow an error of 0.2 cm.
[Notice]
①The foot should be fully extended, the ruler should be loosely clamped to fit, only lightly against the skin, do not have a tight clamp or pressure.
②The foot must be fully stretched and flat when measuring.

23 Biepicondylar humerus breadth

[Definition] The distance between the medial and lateral epicondyles of the humerus.
[Equipment] Digital display Martin ruler (Martin-CHN).
[Preparation] The subject should take off the bracelet or watch, and leave the upper limb bare.
[Method] The subject bends the right upper arm at a right angle to the forearm, and the tester faces the subject and measures the distance between the medial and lateral epicondyles of the humerus with the shoulder width ruler. When recording, the unit is centimeter (cm), retain one decimal, and allow an error of 0.2 cm.
[Notice]
①The tester should accurately find the bony markers of the internal and external humeral condyles.

24 Biepicondylar femur breadth

[Definition] The distance between the medial and lateral epicondyles of the femur above the knee joint.
[Equipment] Digital display Martin ruler (Martin-CHN).
[Preparation] The subject should wear sports shorts with the knee joint exposed.
[Method] The subject bends the left knee at a right angle and the tester measures the distance between the medial and lateral epicondyles of the femur above the knee joint using a shoulder width ruler. When recording, the unit is centimeter (cm), retain one decimal, and allow an error of 0.2 cm.
[Notice]
①The tester should accurately find the bony markers of the internal and external femoral condyles.
