# Supplementary material for: Construction of an anthropometric discriminant model for identification of elite swimmers: an adaptive lasso approach
Source: PeerJ. 2023 Jan 9;11:e14635. doi: 10.7717/peerj.14635 (PMC9835708; doi:10.7717/peerj.14635)

25 Head circumference

[Definition] The circumference of the head from the most prominent point of the superior border of the flush arch through the posterior occipital node.
[Equipment] Soft tape measure.
[Preparation] The subject should not wear a hat and glasses when testing.
[Method] The tester stands in front of or to the right of the subject, and fixes the zero point of the tape measure with the left thumb at the upper edge of the flush arch on the right side of the head; the right hand holds the tape measure counterclockwise through the highest part of the occipital ridge (posterior occipital node) around the head for a week to return to the zero point. When recording, the unit is centimeter (cm), retain one decimal, and allow an error of 0.2 cm.
[Notice]
①The tape measure should be close to the skin when measuring, keeping the horizontal position on the left and right sides, the part with hair should be slightly pressed, and those with long hair should first separate the hair to the top and bottom at the ruler passing.

26 Neck circumference

[Definition] The circumference of the throat node around the level of the neck.
[Equipment] Soft tape measure.
[Preparation] Anything worn around the neck must be removed during the test.
[Method] The tester stands in front of or to the right of the subject, and uses the tape measure with the node of the larynx as the zero point to fix it around the neck level for one week. When recording, the unit is centimeter (cm), retain one decimal, and allow an error of 0.2 cm.
[Notice]
①The tape measure should be loosened and tightened appropriately when measuring, and the horizontal position should be maintained.

27 Elbow circumference

[Definition] The horizontal circumference of the medial superior humeral ankle and ulnar eminence.
[Equipment] Soft tape measure.
[Preparation] The right elbow must be exposed during the test.
[Method] The subject bends the right elbow during the test. The tester uses the tape measure to measure the horizontal circumference of the internal superior humeral ankle and ulnar hawk's beak by passing around the elbow point. When recording, the unit is centimeter (cm), retain one decimal, and allow an error of 0.2 cm.
[Notice]
①The tape measure should be loosened and tightened appropriately when measuring, and the horizontal position should be maintained.

28 Maximum biceps circumference

[Definition] The circumference of the most expanded part of the biceps when the biceps muscle is maximally contracted by clenching the fist and flexing the elbow with force.
[Equipment] Soft tape measure.
[Preparation] The subject should expose the upper arm during the test.
[Method] The subject stands naturally, shoulders relaxed, right arm held obliquely flat at about 45 degrees, palm up and fist and elbow flexion. The tester faces the subject and puts the tape measure around the horizontal circumference of the thickest part of the subject's biceps, and records it in centimeters (cm), retaining one decimal, with an allowable error of 0.2 cm.
[Notice]
①The tape measure should be appropriately tightened when measuring, and the horizontal position should be maintained.
②The subject must make the best effort to flex the elbow with force.

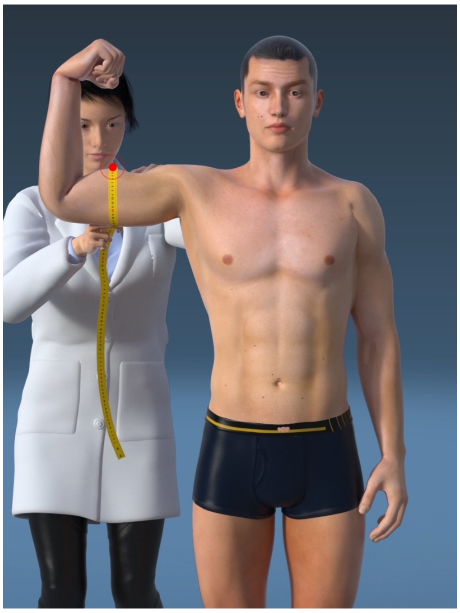

29 Minimum biceps circumference

[Definition] Relaxed circumference of the thickest part of the biceps brachii muscle.
[Equipment] Soft tape measure.
[Preparation] The subject should expose the upper arm during the test.
[Method] After measuring the maximum biceps circumference, make the subject put down the upper limb, relax naturally without moving, and measure the relaxed circumference of the thickest part of the biceps. When recording, the unit is centimeter (cm), retain one decimal, and allow an error of 0.2 cm.
[Notice]
①The tape measure should be loose or tight when measuring, and keep the horizontal position.
②The value can be read only when the upper arm is completely relaxed.

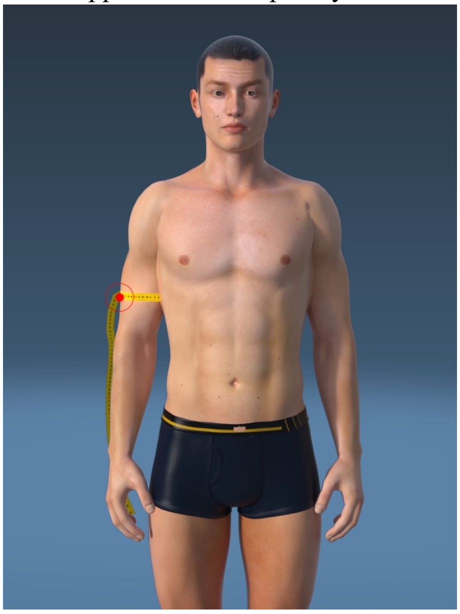

30 Chest circumference

[Definition] The horizontal circumference from the lower edge of the subscapularis to the upper edge of the nipple in men and to the fourth rib above the nipple in women during calm breathing.
[Equipment] Soft tape measure.
[Preparation] The subject's upper body should be naked during the test.

[Method] The subject naturally stands with both feet shoulder-width apart, both shoulders relaxed, both arms hanging naturally, calm breathing state, the tester stands in front of the subject, the upper edge of the tape measure through the lower edge of the back scapula to the upper edge of the nipple in front of the chest, the tape measure around the chest should be suitable for the degree of tightness, in order not to produce significant pressure on the skin as degree. The lower edge of the tape measure is placed at the upper edge of the nipple for men and undeveloped women, and at the fourth thoracic rib joint above the nipple for developed women, and the chest circumference was measured in a calm state, and the value was read at the end of exhalation of the subject, and the value intersecting with the 0 point on the tape measure was the chest circumference value. When recording, the unit is centimeter (cm), retain one decimal, and allow an error of 0.5 cm.

[Notice]

①The measurement is recommended to separate men and women, in a private room, and the upper body should be naked when measuring.

②When the tester conducts the test, pay attention to whether the posture of the subject is correct, whether there is a low head, shrugging shoulders, chest, or hunchback, etc., and correct it in time.

③If the subscapularis cannot be touched, the subject can be made to raise the chest, and the subject should return to the correct testing posture after touching clearly.

④When the subscapularis angle on both sides is not the same, the low side shall prevail.

⑤The tape measure should be loose or tight to maintain the horizontal position.

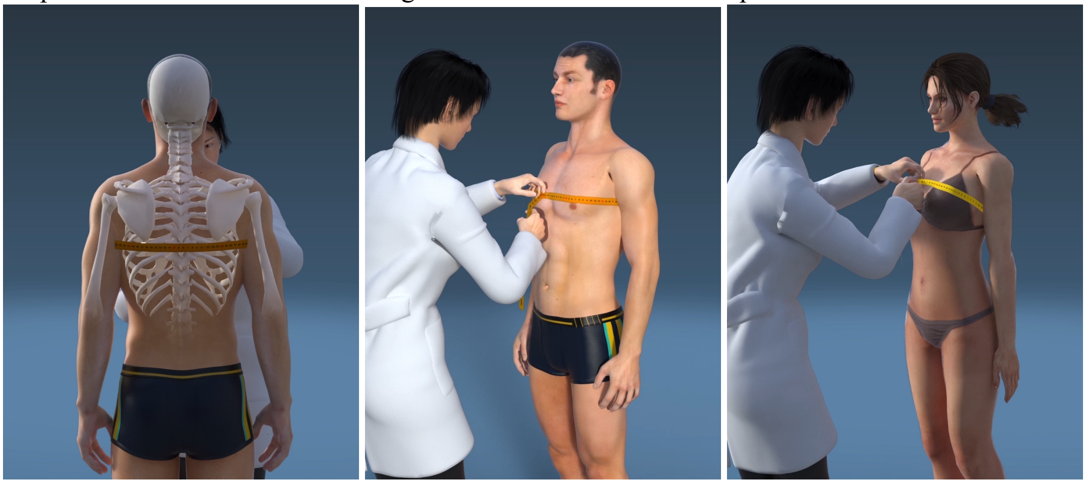

31 Waist circumference

[Definition] The horizontal circumference of the thinnest part of the waist between the rib arch and the iliac crest during calm breathing.

[Equipment] Soft tape measure.

[Preparation] The subject's waist should be completely naked during the test.

[Method] The subject stands straight with feet together, arms hanging naturally, do not tuck in the abdomen, keep breathing steadily. The tester puts the tape measure around the thinnest part of the subject’s waist (between the rib arch and the iliac crest), and measures the horizontal circumference of the thinnest part of the waist during calm breathing. When recording, the unit is centimeter (cm), retain one decimal, and allow an error of 0.5 cm.

[Notice]

①Tester should not produce significant pressure on the subject's skin to avoid large errors.

②When the tape measure is around the waist for a week, it is necessary to pay attention to the section of the tape measure and the body as vertical as possible.

③The tape measure should be loosened and tightened appropriately to maintain a horizontal position.

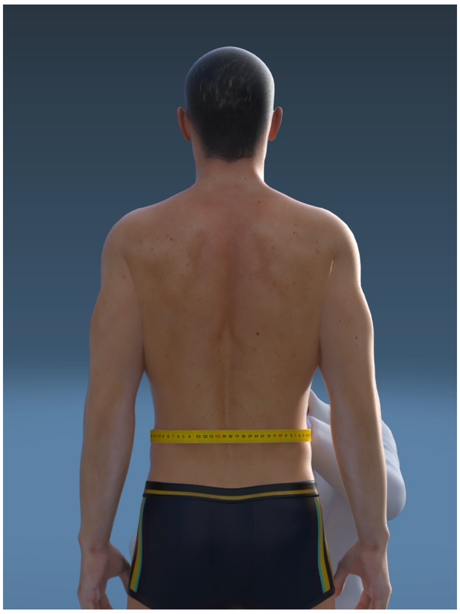

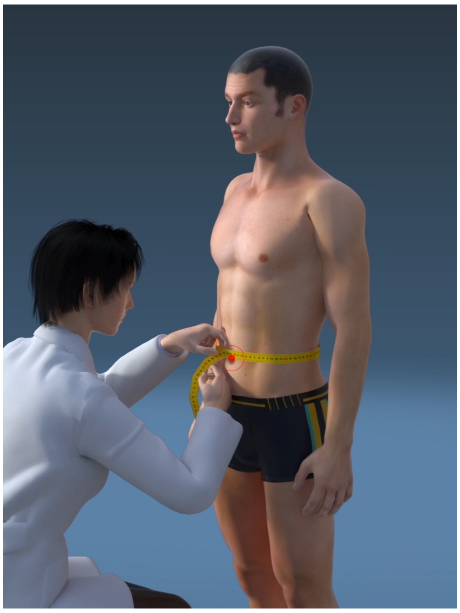

32 Hip circumference

[Definition] The horizontal circumference of the most prominent part of the buttocks toward the back.
[Equipment] Soft tape measure.
[Preparation] The subject should wear close-fitting underwear during the test.
[Method] The subject stands naturally with legs together, arms naturally hanging down and abdomen relaxed. The tester passes the tape measure over the highest point of the buttocks and measures the horizontal circumference of the gluteus maximus at its most prominent point to the pubic symphysis in front (near the root of the thigh). When recording, the unit is centimeter (cm), retain one decimal, and allow an error of 0.5 cm.
[Notice]
①Testers should not produce significant pressure on the subject's skin to avoid large errors.
②When the tape measure is around the buttocks for a week, it is necessary to pay attention to the section of the tape measure and the body as vertical as possible.
③The tape measure should be loosened and tightened appropriately to maintain a horizontal position.

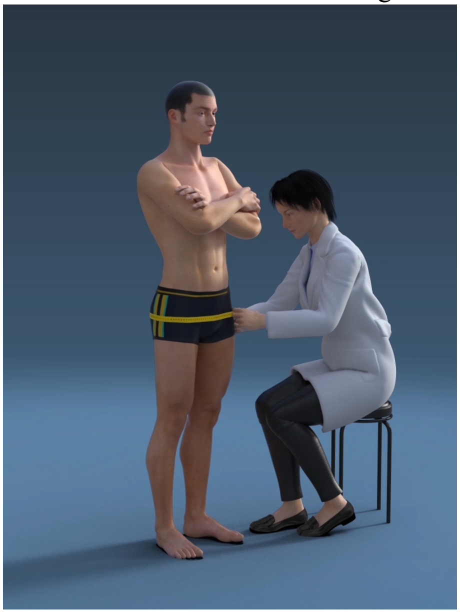

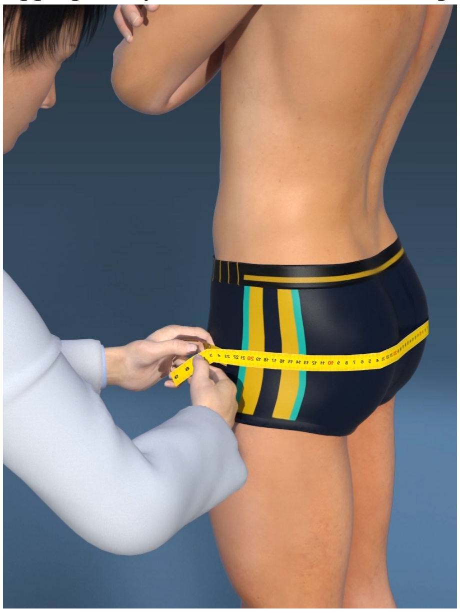

33 Thigh circumference

[Definition] The horizontal circumference of the thickest part of the thigh.
[Equipment] Soft tape measure.
[Preparation] The subject should wear close-fitting underwear during the test.
[Method] The subject stands with both feet shoulder-width apart. The tester stands behind the subject's body side, aligns the upper edge of the tape measure with the root of the thigh, goes around the front horizontally, and measures the horizontal circumference of the thickest part of the thigh. The record was made in centimeters (cm), with one decimal retained, and an error of 0.5 cm is allowed.
[Notice]
①When measuring, the subject's thighs must be kept relaxed and not tight.
②Tester cannot produce significant pressure on the subject's skin, in order to avoid large errors.
③Tape measure elasticity should be appropriate, maintain the horizontal position.

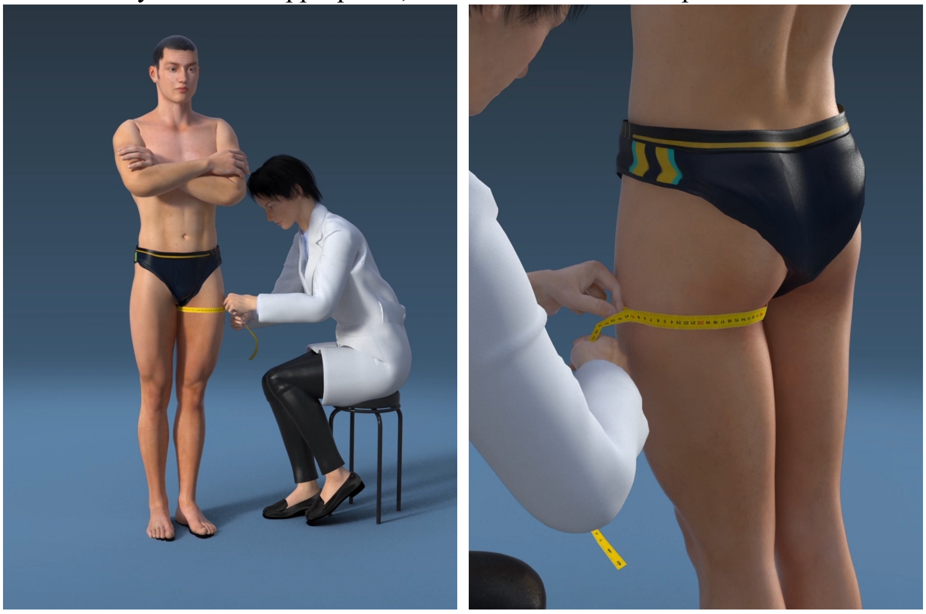

34 Calf circumference

[Definition] The horizontal circumference of the thickest part of the calf gastrocnemius muscle.
[Equipment] Soft tape measure.
[Preparation] The subject should wear sports shorts during the test, exposing the lower leg.
[Method] The subject stands with both feet shoulder-width apart, and both legs are burdened with the average weight. The tester stands on the subject's left side, and measures the circumference of the thickest part of the calf gastrocnemius muscle. The record was made in centimeters (cm), with one decimal retained, and an error of 0.2 cm is allowed.
[Notice]
①When measuring, the subject's calf must be kept relaxed and not tight.
②Testers cannot produce significant pressure on the subject's skin to avoid large errors.
③The tape measure should be loose and tight to maintain the horizontal position.

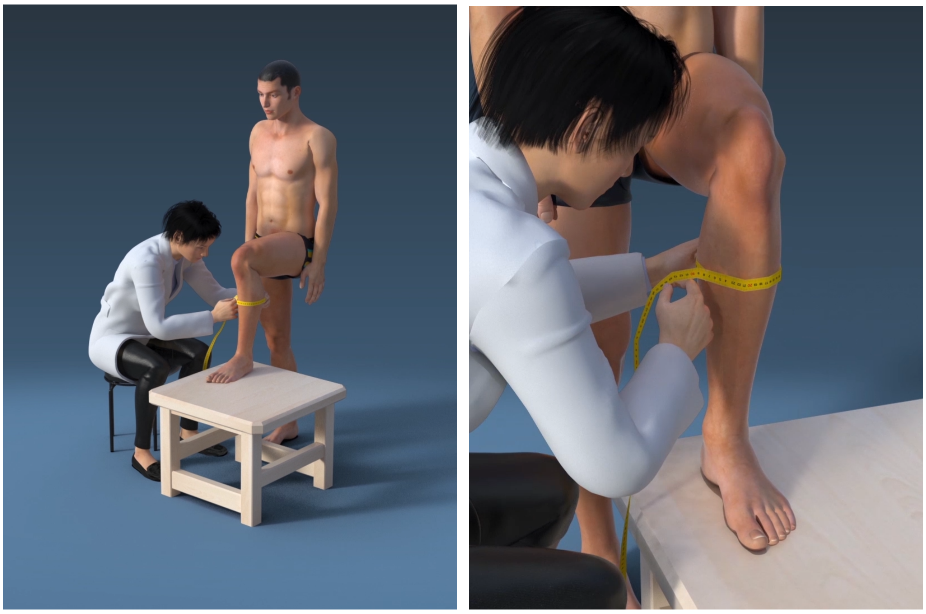

35 Knee circumference

[Definition] The horizontal circumference of the knee through the midpoint of the kneecap.
[Equipment] Soft tape measure.
[Preparation] Subjects should wear shorts with the knee exposed.
[Method] The subject stands with both feet shoulder-width apart, and both legs are burdened with the weight on average. The tester stands in front of the subject's left side and measures the horizontal circumference of the left knee through the midpoint of the kneecap. When recording, the unit is centimeter (cm), retain one decimal, and allow an error of 0.2 cm.
[Notice]
①The subject must remain upright and the tape measure must be kept horizontal during measurement.
②The tape measure should be loose or tight to maintain the horizontal position.

36 Ankle circumference

[Definition] The horizontal circumference of the thinnest point above the ankle joint of the lower leg.
[Equipment] Soft tape measure.
[Preparation] The subject should expose the ankle area during the test.
[Method] The subject naturally stands with both feet shoulder-width apart, and the tester passes the tape measure horizontally around the left calf ankle joint above the inner and outer ankle at the thinnest horizontal position to measure the circumference of its thinnest point. When recording, the unit is centimeter (cm), retain one decimal, and allow an error of 0.2 cm.
[Notice]
①The surrounding part must be the thinnest part of the ankle.
②The tape measure should be loose or tight to maintain the horizontal position.

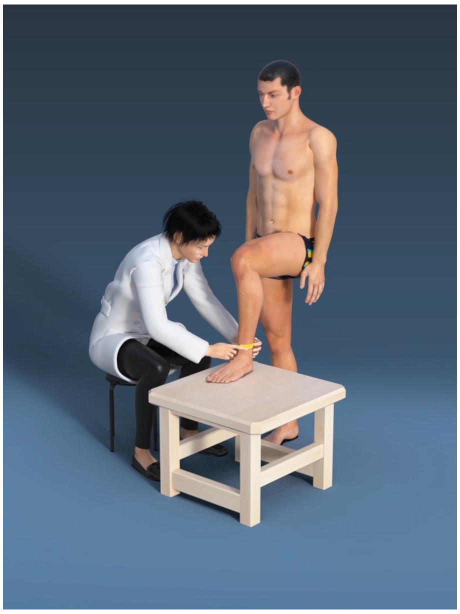

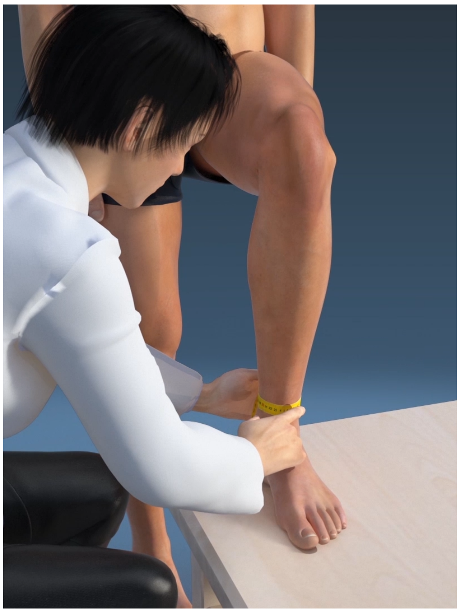

Supplement: Supplemental Information 5 — Includes head circumference, neck circumference, elbow circumference, maximum biceps circumference, minimum biceps circumference, chest circumference, waist circumference, hip circumference, thigh circumference, calf circumference, knee circumference, and ankle circumference. [file peerj-11-14635-s005.pdf]
