# Supplementary material for: Construction of an anthropometric discriminant model for identification of elite swimmers: an adaptive lasso approach
Source: PeerJ. 2023 Jan 9;11:e14635. doi: 10.7717/peerj.14635 (PMC9835708; doi:10.7717/peerj.14635)
Supplement: Supplemental Information 7 [file peerj-11-14635-s007.docx]

**Supplementary Table 1. Competitive level classification (China).**

| **Tier** | **Criteria for classification** |
| --- | --- |
| **International Grade** | Meet one of the following conditions:  a) meet the performance standards in the Olympic Games, Youth Olympic Games, World Championships, Youth World Championships, FINA Swimming World Cup, World University Games, Asian Games, Asian Youth Games, Asian Indoor Games, Asian Championships, Asian Youth Championships, Pan Pacific Swimming Championships, Mediterranean Games, China-Australia Match Race. |
| **National Grade** | Meet one of the following conditions:  a) meet the performance standards in the competitions that can award the International Grade.  b) meet the performance standards in the National Games of the People's Republic of China, Youth Games of the People's Republic of China, National Championships, National Tournaments, National Spring Championships, National Summer Championships, National Youth Championships, National Junior U Series Finals, National Student Games. |
| **1^st^ Grade and 2^nd^ Grade** | Meet one of the following conditions:  a) meet the performance standards in the competitions that can award the National Grade and above.  b) meet the performance standards in the National Traditional Sports Schools League, National Junior U Series, and Games or Championships or Tournaments sponsored by provincial (district or city) sports bureaus. |
| **3^rd^ Grade** | Meet one of the following conditions:  a) meet the performance standards in the competition that can be awarded the 2^nd^ Grade and above.  b) meet the performance standards in the Games or Championships sponsored by city (local, state, league) sports administration department. |
