# Supplementary material for: Construction of an anthropometric discriminant model for identification of elite swimmers: an adaptive lasso approach
Source: PeerJ. 2023 Jan 9;11:e14635. doi: 10.7717/peerj.14635 (PMC9835708; doi:10.7717/peerj.14635)
Supplement: Supplemental Information 8 — There are 36 assessments: 17 for length, 7 for breadth, and 12 for circumference. 28 derivative indexes were calculated, and basic information, such as age of attending the highest level, gender, and competitive level, are also collected, leading to a total of 73 characteristic variables. [file peerj-11-14635-s008.docx]

**Supplementary Table 2. List of the 73 anthropometric variables.**

| **Category** | **n** | **Variables** |
| --- | --- | --- |
| **Basic Information** | 3 | Age of attaining the highest level, Gender, Competitive level |
| **Length**  **(cm)** | 17 | Stature, Sitting height, Span, Upper limb length, Upper arm length, Forearm length, Acromion height, Hand length, Iliospinale posterior height, Trochanterion height, Gluteal height, Eristailiaca height, Thigh length, Lower leg length, Achilles tendon length, Foot length, Back of foot height |
| **Breadth**  **(cm)** | 7 | Biacromial breadth, Biiliocristal breadth, Bitrochanteric breadth, Hand breadth, Foot breadth, Biepicondylar humerus breadth, Biepicondylar femur breadth |
| **Circumference**  **(cm)** | 12 | Head circumference, Neck circumference, Elbow circumference, Maximum biceps circumference, Minimum biceps circumference, Chest circumference, Waist circumference, Hip circumference, Thigh circumference, Calf circumference, Knee circumference, Ankle circumference |
| **Body Composition** | 6 | Weight, Body fat percentage, Fat free mass, Muscle mass, Fat mass, Skeletal muscle mass |
| **Index** | 28 | Quetelet index, Body mass index (BMI), Ratio of sitting height to stature, Relative span, Ratio of arm to stature, Ratio of arm to leg length A, Torso length, Torso length index, Hand area, Foot area, Biceps circumference difference, Ratio of hand breadth to length, Ratio of achilles tendon to lower leg length, Ratio of ankle circumference to achillis tendon length, Ratio of ankle circumference to lower leg length, Ratio of upper to lower leg length, Ratio of leg length A to stature, Leg length A to H, Leg length B to H, Leg length C to H, Ratio of biiliocristal to biacromial breadth, Streamlined index, Ratio of bitrochanteric to biacromial breadth, Ratio of biiliocristal to bitrochanteric breadth, Ratio of waist to chest, Ratio of waist to hip, Pignet Vervaeck index, Ratio of foot breadth to length |

Quetelet index means weight / height * 1000 (g/m); BMI means weight / height^2^ (kg/m^2^); Relative span means span - stature (cm); Arm means upper limb length (cm); Leg length A means iliospinale posterior height (cm); Torso length means acromion height - trochanterion height (cm); Torso length index means torso length / biiliocristal breadth; Hand area means hand length * hand breadth (cm^2^); Foot area means foot length * foot breadth (cm^2^); Biceps circumference difference means maximum biceps circumference - minimum biceps circumference (cm); Upper leg length means thigh length (cm); Leg length H means eristailiaca height (cm); Leg length B means trochanterion height (cm); Leg length C means gluteal height (cm); Streamlined index means (biacromial breadth - biiliocristal breadth) / biiliocristal breadth * stature; Pignet Vervaeck index means (weight + chest circumference) / stature * 100.
