# Supplementary material for: Construction of an anthropometric discriminant model for identification of elite swimmers: an adaptive lasso approach
Source: PeerJ. 2023 Jan 9;11:e14635. doi: 10.7717/peerj.14635 (PMC9835708; doi:10.7717/peerj.14635)
Supplement: Supplemental Information 9 — After the pairwise correlation analysis, a total of 24 candidate variables (23 continuous plus gender) for subsequent analyses. [file peerj-11-14635-s009.docx]

**Supplementary Table 3. Candidate variables for adaptive Lasso**

| **Category** | **n** | **Variables** |
| --- | --- | --- |
| **Basic Information** | 2 | Age of attaining the highest level, Gender |
| **Length**  **(cm)** | 1 | Back of foot height |
| **Circumference**  **(cm)** | 1 | Head circumference |
| **Body Composition** | 2 | Body fat percentage, Skeletal muscle mass |
| **Index** | 18 | Body mass index (BMI), Ratio of arm to stature, Ratio of arm to leg length A, Torso length, Torso length index, Biceps circumference difference, Ratio of hand breadth to length, Ratio of ankle circumference to achillis tendon length, Ratio of upper to lower leg length, Ratio of leg length A to stature, Leg length A to H, Leg length B to H, Leg length C to H, Ratio of bitrochanteric to biacromial breadth, Ratio of biiliocristal to bitrochanteric breadth, Ratio of waist to chest, Ratio of waist to hip, Ratio of foot breadth to length |

BMI means weight / height^2^ (kg/m^2^); Arm means upper limb length (cm); Leg length A means iliospinale posterior height (cm); Torso length means acromion height - trochanterion height (cm); Torso length index means torso length / biiliocristal breadth; Biceps circumference difference means maximum biceps circumference - minimum biceps circumference (cm); Upper leg length means thigh length (cm); Leg length H means eristailiaca height (cm); Leg length B means trochanterion height (cm); Leg length C means gluteal height (cm).
